# The heparan sulfate co-receptor and the concentration of fibroblast growth factor-2 independently elicit different signalling patterns from the fibroblast growth factor receptor

**DOI:** 10.1186/1478-811X-8-14

**Published:** 2010-06-24

**Authors:** Hongyan Zhu, Laurence Duchesne, Philip S Rudland, David G Fernig

**Affiliations:** 1 School of Biological Sciences, Biosciences Building, Crown Street, University of Liverpool, Liverpool, L69 7ZB, UK; 2 Department of Stem Cell Biology, Scientific Park, West China Hospital, Sichuan University, 1 Ke Yuan Si Uu, Gao Peng Da Dao, Chengdu, Sichuan 610041, China; 3 INSERM UMRS 839, Université Pierre et Marie Curie, Institut du Fer a Moulin, 17 rue du Fer a Moulin, 75005 Paris, France

## Abstract

**Background:**

The fibroblast growth factor receptor (FGFR) interprets concentration gradients of FGF ligands and structural changes in the heparan sulfate (HS) co-receptor to generate different cellular responses. However, whether the FGFR generates different signals is not known.

**Results:**

We have previously shown in rat mammary fibroblasts that in cells deficient in sulfation, and so in HS co-receptor, FGF-2 can only stimulate a transient phosphorylation of p42/44 ^MAPK ^and so cannot stimulate DNA synthesis. Here we demonstrate that this is because in the absence of HS, FGF-2 fails to stimulate the phosphorylation of the adaptor FGFR substrate 2 (FRS2). In cells possessing the HS co-receptor, FGF-2 elicits a bell-shaped dose response: optimal concentrations stimulate DNA synthesis, but supramaximal concentrations (≥ 100 ng/mL) have little effect. At optimal concentrations (300 pg/mL) FGF-2 stimulates a sustained dual phosphorylation of p42/44 ^MAPK ^and tyrosine phosphorylation of FRS2. In contrast, 100 ng/mL FGF-2 only stimulates a transient early peak of p42/44 ^MAPK ^phosphorylation and fails to stimulate appreciably the phosphorylation of FRS2 on tyrosine.

**Conclusions:**

These results suggest that the nature of the FGFR signal produced is determined by a combination of the HS co-receptor and the concentration of FGF ligand. Both the phosphorylation of the adaptor FRS2, the kinetics (sustained or transient) of phosphorylation of p42/44(MAPK) are varied, and so differing cellular responses are produced.

## Background

Fibroblast growth factors (FGFs) constitute a family of structurally related proteins, which regulate many facets of cell behaviour, from embryonic patterning, to tissue repair and metabolism [1-3]. FGFs exert their effects on cells by interacting with a signalling receptor tyrosine kinase (FGFR) and a glycosaminoglycan co-receptor, usually heparan sulfate (HS) [4]. Assembly of the complex of FGF ligand, HS and FGFR leads to activation of the intracellular kinase of the receptor through autophosphorylation of two tyrosines in its activation loop, which results in the phosphorylation of other tyrosine residues in the intracellular domain of the receptor that serve as docking sites for SH2 and PTB domain containing proteins [1,5,6]. These include growth factor receptor binder-2 (GRB2) and FGFR substrate 2 (FRS2). FRS2 is a lipid-anchored docking protein, which serves as a major intracellular substrate of the FGFR kinase. Unlike GRB2, which binds via phosphotyrosines [7,8], FRS2 binds to the juxtamembrane region of FGFR via its phosphotyrosine-binding (PTB) domain independently of tyrosine phosphorylation [9]. Upon FGF stimulation, FRS2 is rapidly and highly phosphorylated on multiple tyrosine residues, four of which function as docking sites for the SRC homology2 (SH2) domain of GRB2 [10] and two as binding sites for the N-terminal SH2 domain of the SH2-containing protein tyrosine phosphatase SHP2 [11]. The interaction of SHP2 results in its own tyrosine phosphorylation and complex formation between SHP2 and GRB2. Thus, there are multiple routes for the recruitment of GRB2 to activated FGFR: to phosphorylated tyrosine residues of the FGFR or to phosphorylated tyrosines on FRS2 and SHP2, itself associated with FRS2. The importance of GRB2 recruitment is that it results in the activation of RAS and the downstream activation of mitogen-activated protein kinases (MAPK) p42 and p44. p42/44 ^MAPK ^represent a key pathway in cellular regulation by growth factors and their kinetics of activation can determine cell fate [12].

There is considerable biological evidence to suggest that the FGF receptor-ligand system is able to elicit different signals, depending on the concentration of ligand and the HS co-receptor. In cultured cells FGF ligands elicit a biphasic growth response, such that they stimulate cell proliferation at optimal concentrations, but fail to do so at high concentration [13-15]. *In vivo*, gradients of FGF ligands are a critical component of many developmental events. For example, high and low concentrations of FGFs pattern the ventral foregut into liver and lung [16] and a focal source of FGF is required for limb outgrowth [17]. It is also well established that the HS co-receptor modulates the kinetics of activation of p42/44 ^MAPK ^so that only in the presence of the polysaccharide is the ligand-receptor complex able to elicit signals that lead to cell division [18,19].

However, somewhat surprisingly, it is not known how the HS co-receptor and concentration of FGF ligand may elicit different signals and hence biological outcomes, such as cell division and different cell fates in development. We have, therefore, measured the phosphorylation of both p42/44 ^MAPK ^and the adaptor FRS-2 in cells lacking the HS co-receptor and in cells treated with optimal (300 pg/mL) and high (≥ 100 ng/mL) concentrations of FGF-2. The results indicate that only in the presence of the HS co-receptor can the FGFR phosphorylate the adaptor FRS-2 and so cause the sustained phosphorylation of p42/44 ^MAPK ^necessary for cells to enter the cell cycle. Transient phosphorylation of p42/44 ^MAPK ^can be independent of the phosphorylation of FRS2 and occurs in the absence of the HS co-receptor or at high concentrations of FGF-2.

## Results

### FRS2 phosphorylation depends on HS

**Figure 1:**
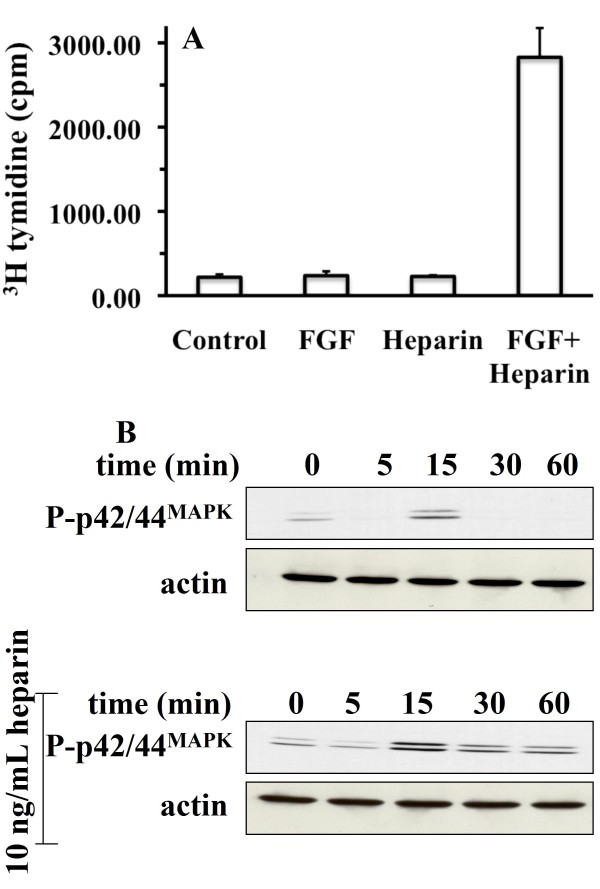
**HS effect on the kinetics of dual phosphorylation of p42/44 **^**MAPK **^(A) DNA synthesis was determined in chlorate-treated quiescent serum-starved Rama 27 fibroblasts by the incorporation of [^3^H] thymidine into DNA 18 h after the addition of growth factor (see Methods), as follows: control, no addition, FGF, (0.3 ng/mL), heparin (10 ng/mL). The results are the mean ± SD of triplicate wells of two experiments. (B) Control or chlorate-treated quiescent Rama 27 fibroblasts were stimulated with 0.3 ng/mL FGF-2 (upper panel) or 0.3 ng/mL FGF-2 and 10 ng/mL heparin (lower panel) for 0 min to 60 min and the doubly phosphorylated Thr ^183/202^/Tyr ^185/204 ^forms of p42/44 ^MAPK ^(P-p42/44 ^MAPK^) were detected with a monoclonal antibody (see Methods). The same blot was re-probed with anti-actin to show the level of loading of the gels.

**Figure 2:**
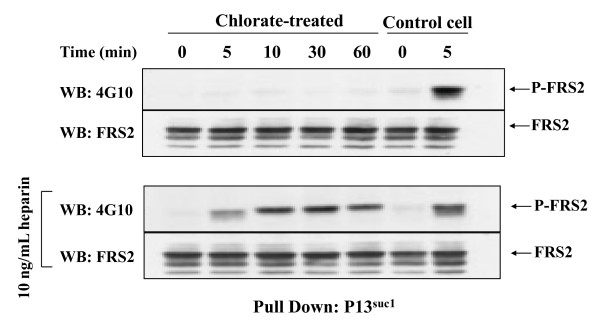
**Effect of HS on FRS2 phosphorylation **Control or chlorate-treated quiescent Rama 27 fibroblasts were stimulated with either 0.3 ng/mL FGF-2 (upper panel) or 0.3 ng/mL FGF2 and 10 ng/mL heparin (lower panel) for 0 min to 60 min. Following pull down with p13 ^suc1^, phosphorylated FRS2 (P-FRS2) was identified by Western blotting (WB) with the phosphotyrosine antibody 4G10 (see Methods). The same blots were re-probed with anti-FRS2 to show the levels of loading of the gel.

The stimulation of DNA synthesis by FGF-2 in rat mammary (Rama) 27 fibroblasts depends on the FGFR activating a sustained dual phosphorylation of p42/44 ^MAPK^. Thus, in chlorate-treated cells, which are HS-deficient, FGF-2 alone only stimulates a transient dual phosphorylation of p42/44 ^MAPK ^and fails to stimulate DNA synthesis. The addition of heparin (a proxy for cellular HS) restores the stimulation of DNA synthesis and the dual phosphorylation of p42/44 ^MAPK ^is sustained (Fig. 1), as we have shown previously [18,19]. A key question is how might the HS co-receptor influence the kinetics of phosphorylation of p42/44 ^MAPK^. Since the phosphorylation of FRS-2 is a key early event in FGF signalling, this was measured. Interestingly, in cells rendered deficient in sulfated HS by treatment with chlorate, stimulation by FGF-2 failed to cause the phosphorylation of FRS2. In contrast, in the presence of heparin in the same chlorate-treated cells, FRS2 phosphorylation was detected 5 min after the addition of FGF-2 and its phosphorylation was sustained to the end of the experiment (60 min, Fig. 2).

### Dose response of FGF-2 stimulation of DNA synthesis and of dual phosphorylation of p42/44 ^MAPK ^in Rama 27 fibroblasts

**Figure 3:**
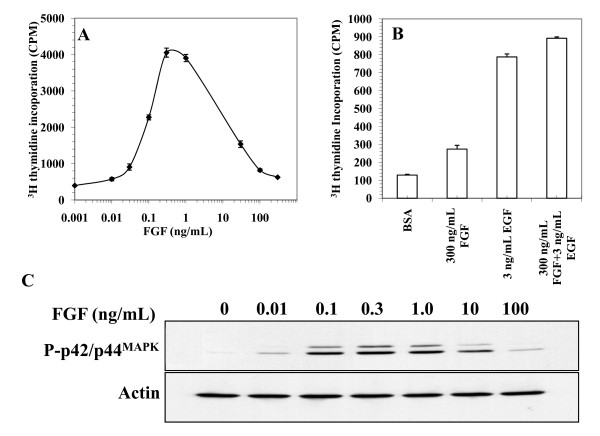
**Dose-response of DNA synthesis and dual phosphorylation of p42/44 **^**MAPK **^**stimulated by FGF-2 **Quiescent serum-starved Rama 27 fibroblasts were stimulated with FGF-2 and EGF. (A, B) DNA synthesis was determined by the incorporation of [^3^H] thymidine into the DNA of quiescent Rama 27 fibroblasts 18 h after the addition of growth factor (see Methods) and results are the mean ± SD of triplicate wells of two experiments. (A) Dose response curve for FGF-2. (B) Stimulatory effect of 3 ng/mL EGF in the absence or presence of 300 ng/mL FGF-2. BSA is the negative control with no growth factor. (C) Dose-response of dual phosphorylation of p42/44 ^MAPK ^in quiescent Rama 27 fibroblasts stimulated with the indicated doses of FGF-2 for 15 min. The doubly phosphorylated Thr ^183/202^/Tyr ^185/204 ^forms of p42/44 ^MAPK ^(P- p42/44 ^MAPK^) were detected with a monoclonal antibody (see Methods). The same blot was re-probed with anti-actin to show the level of loading of the gel.

Thus, the above data indicate that the HS co-receptor is essential for the FGF-2 activated FGFR to phosphorylate FRS-2, which is itself central to the sustained phosphorylation of p42/44 ^MAPK^. Intrigued by how changing the extracellular composition of the signalling complex (FGF-2 + HS + FGFR *versus *FGF + FGFR) determined whether the adaptor FRS-2 was phosphorylated, we explored another parameter that from a biological perspective seemed likely to alter signalling, the concentration of FGF-2 ligand. The stimulation of DNA synthesis in Rama 27 fibroblasts was determined over an extended range of concentrations of FGF-2 (0.01 ng/mL to 300 ng/mL). As previously shown in these cells [20], a stimulation of DNA synthesis was first observed at 0.01 ng/mL FGF-2 and reached a maximum level at 0.3 ng/mL to 1 ng/mL FGF-2 (Fig 3A). However, when the concentration of FGF-2 was increased further, the level of stimulation of DNA synthesis declined from this maximal level to reach near basal levels at 300 ng/mL FGF-2 (Fig. 3A). As is common in cell dose responses, some variation in the downward curve was observed, which is reflected by the magnitude of the residual stimulation of DNA synthesis observed at 300 ng/mL FGF-2 (Fig. 3A, B). The low level of stimulation of DNA synthesis at high concentrations of FGF-2 is likely to be due to a specific change in FGF receptor signalling, since when 3 ng/mL EGF was added together with 300 ng/mL FGF-2, the level of DNA synthesis was similar to that observed with EGF alone (Fig. 3B). These results demonstrate that FGF-2 elicits a bell-shaped dose response in Rama 27 fibroblasts, similar to that observed by others in human umbilical endothelial cells [13]. Importantly, high concentrations of FGF-2 do not inhibit other growth factors from stimulating DNA synthesis. We next examined the dual phosphorylation of p42/44 ^MAPK ^in Rama 27 fibroblasts in response to different concentrations of FGF-2. Fifteen minutes after the addition of the growth factor, a small increase in the dual phosphorylation of p42/44 ^MAPK ^was observed in cells stimulated with 0.01 ng/mL FGF-2 and this reached a maximum at 0.3 ng/mL FGF-2 (Fig. 3C). At higher concentrations of the growth factor, the dual phosphorylation of p42/44 ^MAPK ^declined from this maximal level such that 100 ng/mL FGF-2 elicited the same low level of phosphorylation of p42/44 ^MAPK ^as 0.01 ng/mL FGF-2. Therefore, the concentration dependence of FGF-2 induced p42/44 ^MAPK ^phosphorylation (Fig. 3C) followed a similar bell shape to that observed for DNA synthesis (Fig. 3A).

### Kinetics of p42/44 ^MAPK ^phosphorylation induced by different doses of FGF-2

**Figure 4:**
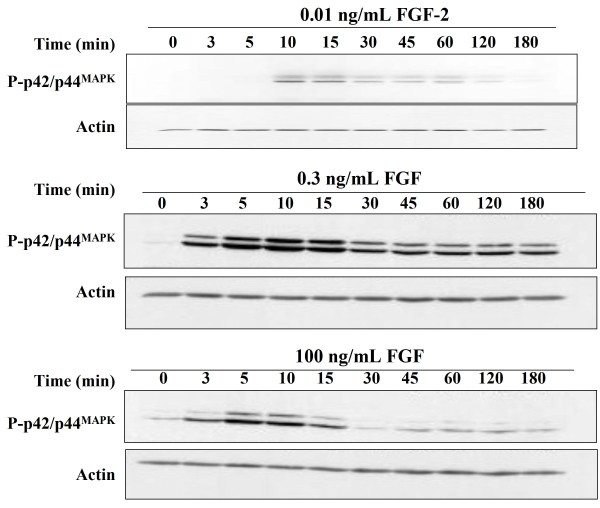
**Kinetics of dual phosphorylation of p42/44 **^**MAPK **^**induced by different concentrations of FGF-2 **Quiescent Rama 27 fibroblasts were stimulated with the indicated doses of FGF-2 for 0 min to 180 min. The doubly phosphorylated Thr ^183/202^/Tyr ^185/204 ^forms of p42/44 ^MAPK ^(P- p42/44 ^MAPK^) were detected with a monoclonal antibody (see Methods). The blots were re-probed with anti-actin to show the level of loading of the gels.

One simple explanation for the decrease in p42/44 ^MAPK ^phosphorylation observed as the concentration of FGF-2 was increased from 0.3 ng/mL to 100 ng/mL was that higher concentrations of growth factor would initiate robust signalling earlier compared to lower concentrations of FGF-2. These signals might also be inhibited earlier by negative feedback loops. If this was the case, the maximal p42/44 ^MAPK ^phosphorylation induced by the highest concentration of FGF-2 should occur earlier and more strongly than that found with lower concentrations of the growth factor. To address this possibility, we investigated the kinetics of p42/44 ^MAPK ^phosphorylation in response to 0.01 ng/mL, 0.3 ng/mL and 100 ng/mL FGF-2. Following the addition of 0.3 ng/mL FGF-2, the dual phosphorylation of p42/44 ^MAPK ^was detected within 3 min and reached a maximal level at 10 min. Subsequently, 30 min after the addition of FGF-2, the dual phosphorylation of p42/44 ^MAPK ^decreased quite sharply to a lower level, which was maintained to the end of experiment (Fig 4). The kinetics of p42/44 ^MAPK ^phosphorylation following the addition of 0.01 ng/mL FGF-2 showed a similar pattern to that stimulated by 0.3 ng/mL. However, the onset of the response was delayed and its amplitude was weaker. Moreover, the plateau that followed the initial peak in phosphorylation of p42/44 ^MAPK ^declined gradually to nearly undetectable levels by the end of the experiment (Fig. 4). These differences might be expected from a submaximal stimulus, which is only effective at stimulating a minority of the cells into S-phase of the cell cycle (Fig. 3A). In response to the addition of 100 ng/mL FGF-2, the phosphorylation of p42/44 ^MAPK ^was again apparent at 3 min and reached a maximum at 5 min. However, the level of phosphorylation of p42/44 ^MAPK ^declined by 15 min and reached near basal levels at 30 min (Fig. 4). Moreover, the amplitude of the early peak of p42/44 ^MAPK ^phosphorylation was reduced when cells were stimulated with this high concentration of FGF-2 compared to cells which were stimulated with 0.3 ng/mL FGF-2.

### Effects of FGF-2 concentration on FRS2 phosphorylation

**Figure 5:**
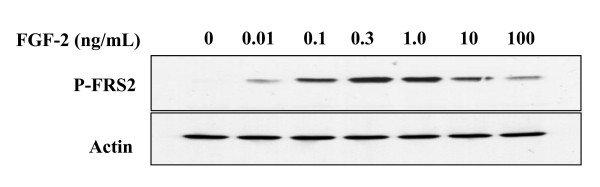
**FRS2 phosphorylation induced by FGF-2 **Quiescent Rama 27 fibroblasts were stimulated with the indicated doses of FGF-2 for 5 min. Phosphorylated FRS2 was identified by western blotting with phospho-FRS2-α (Tyr196) Antibody (see Methods). The same blot was re-probed with anti-actin to show the level of loading of the gel.

It was of interest to determine whether the different kinetics of p42/44 ^MAPK ^phosphorylation were associated with differences in the phosphorylation of FRS-2. To this end the phosphorylation of FRS2 was initially examined 5 min after stimulation of cells with different concentrations of FGF-2. Consistent with the observed concentration-dependence of DNA synthesis and p42/44 ^MAPK ^phosphorylation, the maximum level of tyrosine phosphorylation of FRS2, detected as an immunoreactive band of around 90 kDa with an antibody to phospho-FRS2-α (Tyr196), also occurred at 0.3 ng/mL of FGF-2 (Fig 5). At 0.01 ng/mL and 100 ng/mL of FGF-2 a much lower level of phosphorylation of FRS2 was observed, which was only just above background (Fig. 5). This result showed that phosphorylation of FRS2 was also dependent on the concentration of FGF-2 and displayed a bell-shaped dose-response curve.

**Figure 6:**
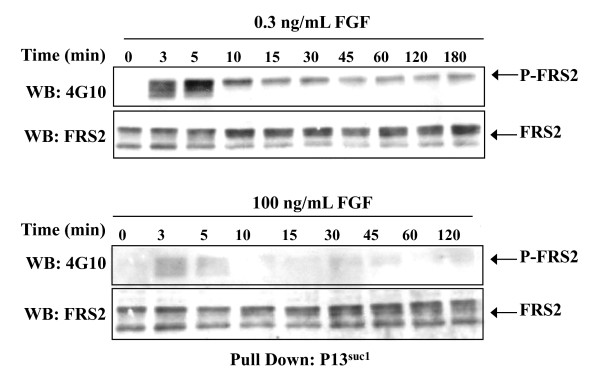
**Kinetics of FRS2 phosphorylation induced by different concentration of FGF-2 **Rama 27 fibroblasts were stimulated with the indicated doses of FGF-2 for 0 min to 180 min. Following pull down with p13 ^suc1^, phosphorylated FRS2 was identified by Western blotting (WB) with the phosphotyrosine antibody 4G10 (see Methods). The same blots were re-probed with anti-FRS2 to show the levels of loading. Upper panel 0.3 ng/mL FGF-2; lower panel 100 ng/mL FGF-2.

It was important to measure the kinetics of phosphorylation of FRS2 to determine whether 100 ng/mL FGF-2 may elicit an earlier or a later peak of phosphorylation of FRS2, compared to that occurring in the presence of 0.3 ng/mL FGF-2. In addition, since the phosphospecific antibody only detects phosphorylation of one of several tyrosines in FRS2, pull down with p13 ^SUC1 ^followed by immunoblotting with an antibody to phosphotyrosine was used. In this way we could be certain that a lack of signal was not due to a shift in the phosphorylation of specific tyrosines in FRS2. Phosphorylation of FRS2 on tyrosine was detected at 3 min after the addition of 0.3 ng/mL FGF-2 and reached a maximal level after 5 min. The phosphorylation of FRS2 then declined rapidly at 10 min to reach a plateau at 15 min to 45 min, which was sustained to the end of the experiment, 180 min (Fig. 6). However, in response to 100 ng/mL FGF-2 only a very low level of FRS2 phosphorylation was detected between 3 min and 5 min and this level then declined by 10 min to the basal levels observed in the unstimulated cells (Fig 6).

## Discussion

A few studies carried out in the late 1980s evaluated the activity of purified FGFs over a wide concentration range and their results show that the stimulation of cell proliferation follows a bell-shaped dose response. Thus, cell proliferation stimulated by FGF-2 in human umbilical vein endothelial cells has a biphasic dose response, such that supramaximal concentrations of the growth factor result in submaximal to basal DNA synthesis [13]. Similar results have been obtained with FGF-1 in Balb/c 3T3 fibroblasts [15] and in Rama 27 fibroblasts [14]. The present results show that FGF-2 in Rama 27 fibroblasts also elicits a bell-shaped dose response. It seems reasonable to suggest that such bell-shaped dose response curves are a general property of at least FGF-1 and FGF-2, if not all FGFs. Importantly, the low to basal stimulation of DNA synthesis at high concentrations of FGF-2 is intrinsic to FGF-2, since EGF still stimulates DNA synthesis when FGF-2 is also present at high concentration (Fig 3). It should be noted that the above work uses S-phase entry as a readout of the activity of the FGF. *In vivo *FGFs stimulate not just cell proliferation, but also cell survival, differentiation, migration and metabolism [1]. Therefore, the observed lack of cell proliferation observed at high concentrations of the growth factor does not mean that the FGF is without biological effect in healthy tissue, as well as in disease. Indeed, the biphasic response of cells to FGF-1, FGF-2 and perhaps all FGFs has biological implications, which is reflected by experiments *in vivo *. Tissues can contain high levels of FGFs. Thus, in the virgin rat mammary gland there is 70 ng/g wet weight of extractable FGF activity [21]. Concentration gradients of FGFs are a key feature of organogenesis. For example, in limb development the creation and maintenance of a concentration gradient of FGFs is crucial for the function of the apical ectodermal ridge in promoting limb outgrowth, since only a focal concentration of FGFs can replace the apical ectodermal ridge, whereas exposure of the entire embryo to FGFs will not result in additional limb development [17]. Similarly, high and low concentrations of FGFs pattern the ventral foregut into liver and lung [16]. These *in vivo *observations indicate that the FGFR should be able to elicit different intracellular signals, dependent on the concentration of FGF ligand.

To understand why FGF-2 at high concentrations fails to stimulate DNA synthesis, two important signalling events downstream of the FGFR were examined, the phosphorylation of the adaptor FRS2 and of p42/44 ^MAPK^. The phosphorylation of p42/44 ^MAPK ^is the result of the activation of the GRB2, RAS-RAF-MEK pathway and is essential for the stimulation of DNA synthesis in these cells. Recruitment of GRB2 can occur directly via the FGFR or indirectly via the adaptor FRS2. At optimal concentrations for DNA synthesis, FGF-2 stimulated a sustained phosphorylation of FRS2 and of p42/44 ^MAPK^. At high concentrations of FGF-2, the adaptor protein FRS2 is only very weakly phosphorylated for a short time, whereas the transient early phosphorylation of p42/44 ^MAPK ^was substantial. The transient very weak phosphorylation of FRS2 may be due simply to the time taken for the ligand-receptor system to reach equilibrium with the exogenous 100 ng/mL FGF-2. At 4°C it takes 60 min to 90 min for the binding of exogenous FGF-2 to reach equilibrium in these cells [20] and at 37°C equilibration will be faster by about 6.5 fold, due to the increased thermal energy. Thus, high concentrations of FGF-2 fail to result in appreciable phosphorylation of FRS2, but still stimulate a transient phosphorylation of p42/44 ^MAPK^, which is likely to result from the recruitment of GRB2 to the FGFR. These observations suggest that phosphorylation of FRS2 and consequent recruitment of GRB-2 via FRS2 is essential for a sustained downstream phosphorylation of p42/44 ^MAPK^, whereas recruitment of GRB2 directly to the FGFR results only in a transient phosphorylation of p42/44 ^MAPK^.

Using a different approach with chlorate-treated, HS-deficient cells, signalling assemblies lacking the HS co-receptor and consisting of FGF2 ligand and FGFR were examined. The results show that phosphorylation of FRS2 again determines the kinetics of phosphorylation of p42/44 ^MAPK^. In the absence of HS, there is no detectable phosphorylation of FRS2, only a transient phosphorylation of p42/44 ^MAPK ^is observed and DNA synthesis does not occur.

Other work has indicated that FRS2 is central to the FGFR eliciting a sustained phosphorylation of p42/44 ^MAPK^. In cells expressing mutant FGFR-1, which cannot bind GRB2 directly, since tyrosine residues lying outside the activation loop that are normally phosphorylated are mutated to phenylalanine, FGF-1 still stimulates phosphorylation of FRS2, activation of the GRB2-p42/44 ^MAPK ^pathway and cell proliferation [7]. This mutant receptor can now only recruit GRB2 indirectly through FRS2 and SHP2. In embryonic fibroblasts isolated from *frs2 *null mice, FGF-1 can only cause the recruitment of GRB2 directly to the FGFR, which results in a transient phosphorylation of p42/44 ^MAPK ^and no stimulation of cell proliferation; re-expression of FRS2 in the *frs2 *null cells restores the ability of FGF-1 to stimulate a sustained phosphorylation of p42/44 ^MAPK ^and cell proliferation [22].

An important question is how might changes in FGF-2 concentration or the absence of HS co-receptor cause the altered phosphorylation kinetics of FRS2 and p42/44 ^MAPK ^and so change the biological outcome of FGF signaling. In the case of the HS co-receptor, it is now established that FGF ligands can engage the FGFR independently of the polysaccharide [23]. Thus, one explanation for the present results is that only in the presence of HS can FGF-2 cause the assembly of a FGFR complex that is capable of phosphorylating FRS2.

In the case of the concentration of FGF ligand, the most likely explanations are either changes in negative feedback loops that depend on ligand concentration or differences in the type of receptor complex that is assembled. Negative feedback that could be increased at high concentrations of FGF-2 includes dephosphorylation and receptor down-regulation. Although neither can be formally excluded, three lines of evidence suggest that negative feedback may be a contributory, but not a dominant mechanism: the lack of phosphorylation of FRS2 at high concentrations of FGF-2, which is observed at times too short for receptor down-regulation; the differences in timing of the first peak of p42/44 ^MAPK ^phosphorylation correlates with concentration and in all likelihood reflects increased on-rates of FGF-2 ligand; the reduced amplitude of the first peak of p42/44 ^MAPK ^phosphorylation observed with high, compared to optimal concentrations of FGF-2.

The assembly of the FGF receptor ligand system could change due to the concentration of FGF-2, particularly since there is evidence for FGF-2 possessing two binding sites for the FGFR of different affinities [24] and three binding sites for the HS co-receptor, which are also likely to be of different affinity [25]. Consequently, interactions of FGF-2 with HS or the FGFR that may be of high stoichiometry, e.g., 1FGF:2FGFR, at low concentrations of FGF-2, would be of lower order stoichiometry, e.g., 1FGF:1FGFR, at the high concentration of the growth factor used here; 300 pg/mL or 16 pM is around the K _D _of the high affinity binding site on these cells, whereas 100 ng/mL or 5.6 nM is more than two orders of magnitude higher [20]. This may enable the secondary binding sites on the FGF-2 ligand to engage their partner(s) independently of the primary binding site. The observation that FRS2 is only phosphorylated at lower concentrations of FGF-2 indicates that this event may depend, in turn, on an extracellular interaction of the FGF-2 that has a high stoichiometry. Given that biophysical and structural analyses have produced competing views of the assembly of the FGF ligand-receptor complex [24,26-30], it may be that more than one of these is physiologically relevant, depending on the conditions. However, such *in vitro *analyses do not account for the complexity of interactions at the cell surface. For example, there is good evidence for neuropilin [31], anosmin-1 [32], cell adhesion molecules such as L1 and cadherins [33-36] and integrins [37] not only binding directly to one or more of the components of the FGF-receptor complex, but also modulating the activity of the FGFR. Thus, there remains a substantial challenge in defining the FGF receptor-ligand complex(es) that are formed on living cells and responsible for the different types of signalling observed in the present work.

## Conclusions

The results show that the concentration of FGF ligand and the presence of HS co-receptor determine whether the FGFR phosphorylates the adaptor FRS2. This determines the kinetics of phosphorylation of p42/44 ^MAPK^, which in turn decides whether FGF signaling stimulates cell proliferation or a different response. These data demonstrate that the FGF receptor ligand system is capable of generating distinct signal outputs due to parameters (HS and FGF ligand concentration) that are known to determine FGF signaling in development and may be important in disease. Therefore the results demonstrate that these parameters can switch FGF signaling between a proliferative and a non-proliferative response and this would then enable the FGFR to interpret FGF gradients in development [16,17] and differences in HS structures[38].

## Methods

### Materials

Human recombinant FGF-2 was obtained from R & D Systems (Abingdon, Oxon, UK) and mouse epidermal growth factor (EGF) was obtained from Pepsyn (Liverpool, UK). Cell culture reagents were from Life Technologies (Paisley, UK). Reagents for SDS-PAGE and electrotransfer were purchased from Bio-Rad (Hemel Hempstead, Herts, UK). Protease inhibitor mixture was from Roche Molecular Biochemicals (Lewes, UK). Antibody to phosphotyrosine (4G10) and P13 ^suc1 ^agarose were from UpState Biotechnologies (Milton Keynes, UK). Anti-FRS2 was from Santa Cruz Biotechnology (Heidelberg, Germany). Anti- p42/44 ^MAPK^, anti-phospho-p44/42 ^MAPK ^(Thr ^183/202^/Tyr ^185/204^) (E10) and anti-phosphoFRS2-α (Tyr 196) were from Cell Signalling Technology (Hitchin, UK). Anti-actin was from Sigma-Aldrich Co. Secondary peroxidase-labelled anti-IgG antibodies (anti-rabbit and anti-mouse) were from Amersham Bioscience (Little Chalfont, Bucks., UK).

### Cell Culture and DNA Synthesis

Rama 27 fibroblasts were cultured in Dulbecco's modified Eagle's medium (DMEM) supplemented with 5% (v/v) fetal calf serum, 50 ng/mL insulin and 50 ng/mL hydrocortisone [39]. Chlorate was used to inhibit sulfation of glycosaminoglycans, by virtue of its inhibition of the synthesis of PAPS (3'-phosphoadenosine 5'-phosphosulfate), the activated sulfate donor [40-42]. Sulfated glycosaminoglycan-deficient Rama 27 cells were prepared as described [43]. Concisely, cells were incubated for 4 h in sulfate-free Dulbecco's modified Eagle's medium, supplemented with 10% (v/v) dialyzed fetal calf serum and 15 mM NaClO _3_. Following trypsinisation, the cells were seeded in plates as appropriate for the measurement (DNA synthesis, Western blotting, pull down), except that sulfate-free Dulbecco's modified Eagle's medium supplemented with 15 mM NaClO _3 _was used throughout.

DNA synthesis assays were performed as described previously [18,19]. Rama 27 fibroblasts, seeded in 24-well plates at a density of 15000 cells/well were rendered quiescent by 24 h incubation in 500 μL serum-free DMEM containing 250 μg/mL bovine serum albumin (step down medium-SDM). The medium was replaced with fresh SDM 6 h before the addition of growth factors. Twenty μL of 40 μCi/mL [methyl- ^3^H] thymidine (ICN, Basingstoke, UK) was added directly to the culture medium 18 h later for 1 h and radioactivity in DNA, precipitated with 5% (w/v) trichloroacetic acid, was measured by liquid scintillation counting.

### P13 ^suc1 ^Pull Down

FRS2 binds the cyclin-dependent kinase substrate p13 ^suc1 ^[44], which provides a convenient means to extract FRS2 from cell lysates. Rama 27 fibroblasts were seeded at 20,000 cells/cm ^2 ^in 9 cm diameter culture dishes (1.3 × 10 ^6 ^cells in 10 mL medium per dish) and then treated as for the DNA synthesis assay up to the addition of growth factors. At the times indicated, Rama 27 cells were washed twice with ice-cold Tris-buffered saline (TBS) contained 100 μM sodium vanadate and then lysed in 500 μL lysis buffer (20 mM Tris-HCl, 137 mM NaCl, 10% (v/v) glycerol, 1% (v/v) NP-40, 1 mM sodium vanadate, pH7.0) with one tablet of protease inhibitor mixture (Roche, Lewes, UK) per 10 mL. Culture dishes were gently rocked for 20 min at 4°C after addition of lysis buffer. Adherent material was then detached by scraping and the lysates were transferred to pre-cooled microcentrifuge tubes. Culture dishes were washed once with 200 μL of lysis buffer, which was combined with the lysate. Lysates were clarified by centrifugation at 12000 g for 10 min. The supernatants were then transferred to fresh microcentrifuge tubes containing 40 μL lysis buffer and p13 ^suc1 ^agarose suspension (3:1 v/v) and the slurry was incubated at 4°C on a rocker platform overnight. Agarose beads were collected by centrifugation at 12000 g for 5 min and washed by centrifugation three times with lysis buffer and once with water. Following resuspension of the final pellet in 60 μL electrophoresis sample buffer (250 mM Tris-HCl, 1% (w/v) SDS, 0.006% (w/v) bromophenol blue, 2% (v/v), β-mercaptoethanol, pH 6.8) with one tablet of protease inhibitor mixture per 10 mL, samples were boiled for 5 min.

### Identification of MAPK and FRS2 phosphorylation

Rama 27 fibroblasts were cultured exactly as for p13 ^suc1 ^pull-down experiments. At the times indicated, Rama 27 cells were washed twice with ice-cold PBS and collected by scraping in 300 μL of 2× electrophoresis sample buffer and then boiled for 5 min.

### SDS-PAGE and Western Blotting

Polypeptides in samples containing identical amounts of protein were separated by electrophoresis on appropriate polyacrylamide gels in the presence of SDS and transferred to nitrocellulose membranes. Following blocking with TBS containing 0.05% (v/v) Tween 20 (TBS-Tween) and 5% (w/v) non-fat dry milk (blocking buffer) for 1 h, membranes were probed with appropriate primary antibodies overnight with gentle shaking at 4°C. After three washes with TBS-Tween, the membranes were incubated with the corresponding secondary peroxidase-conjugated polyclonal antibodies in blocking buffer for 1 h, washed three times in TBS-Tween, and bound peroxidase was detected with the SuperSignal chemiluminescent system (Pierce and Warriner, Chester, UK) on Hyperfilm (Amersham Pharmacia Biotech). Activated p42/44 ^MAPK ^was detected using a monoclonal antibody directed against the doubly phosphorylated Thr ^183/202^Tyr ^185/204 ^forms of these enzymes. This antibody does not cross-react, according to the manufacturer, with the corresponding doubly phosphorylated SAPK/JNK, p38 MAPKs or monophosphorylated p42/44 ^MAPK^. The phosphorylation of FRS2 was detected using anti-phosphotyrosine 4G10 following P13 ^suc1 ^pulling down or by a polyclonal antibody directed against the phosphorylated FRS2-α (Tyr196). This antibody does not cross-react with unrelated tyrosine-phosphorylated proteins and it produced results equivalent to the pull down followed by Western blotting with 4G10. Where appropriate, the same membrane was re-probed with anti-actin or anti-FRS2 to show the level of loading. Each Western blot is one of at least three independent experiments.

## Competing interests

The authors declare that they have no competing interests.

## Authors' contributions

HZ performed the bulk of the experiments, with LD assisting. DGF and PSR conceived the idea for this study, HZ and DGF wrote the manuscript. All authors read and approved the submitted manuscript.
